# Dr. Hulusi Behçet: A Pioneer in Dermatology and the Legacy of Behçet's Disease

**DOI:** 10.7759/cureus.69600

**Published:** 2024-09-17

**Authors:** Gizem Ecem Kocak Nuhoglu, Cagatay Nuhoglu, Merve Osoydan, Banu Arslan

**Affiliations:** 1 Oral and Maxillofacial Surgery, Istanbul Kent University, Istanbul, TUR; 2 Emergency Medicine, Sisli Hamidiye Etfal Training and Research Hospital, Istanbul, TUR; 3 Emergency Medicine, Başakşehir Çam and Sakura City Hospital, Istanbul, TUR

**Keywords:** autoimmune diseases, behcet’s syndrome, historical vignette, hulusi behcet, morbus behcet

## Abstract

Dr. Hulusi Behçet (1889-1948) was a renowned Turkish dermatologist who made significant contributions in the field. His innovative research led to the discovery of Behçet's disease, a complex autoimmune disorder characterised by recurring mouth ulcers, inflammation of the eye, genital ulcers, and skin lesions. Behçet was born in Istanbul and had a comprehensive medical education, which he expanded through subsequent studies in Europe. His medical career was marked by his astute observations and rigorous study, ultimately leading to the official identification of Behçet's disease in 1937. Behçet wrote more than 100 publications and made substantial contributions to the fields of dermatology and internal medicine, particularly in the areas of syphilis and cutaneous leishmaniasis research. The impact of his work may still be seen in the ongoing investigation and management of Behçet's illness, leading to enhanced well-being for numerous individuals across the globe. Dr. Behçet's commitment to medical science is honoured by the recognition of Behçet's disease, academic accolades, and institutions named after him. His work continues to be a fundamental aspect in the examination of autoimmune illnesses, demonstrating the lasting influence of his contributions to the field of medical science.

## Introduction and background

Dr. Hulusi Behçet (1889-1948) was a pioneering Turkish dermatologist and researcher renowned for his contributions to understanding and identifying Behçet's disease, a complex autoimmune condition. Hulusi Behçet commenced his endeavours during the final stages of the Ottoman Empire, a time when the nation was engaged in warfare. Subsequently, he proceeded with his efforts in Turkey, a nation fatigued from a significant conflict and constrained in its prospects. Throughout this period, he frequently travelled abroad and honed his expertise in dermatology. He has accomplished numerous groundbreaking achievements in the field of dermatology in the recently founded country of Türkiye and has actively contributed to the education of future generations [1]. His work laid the groundwork for a significant field of medical research and has had a lasting impact on dermatology and internal medicine.

## Review

Early life and education

Hulusi Behçet was born in Istanbul on February 20, 1889, then part of the Ottoman Empire. He came from a family with a background in education and medicine, which influenced his decision to pursue a medical career. Hulusi Behçet lost his mother at an early age. This situation made him a little more introverted [1]. Despite the difficulties he experienced in his childhood, he received a good education and learned French, Latin, and German. He was only 21 when he graduated from Gülhane Military Medical Academy. When he finished his speciality in dermatology, the date was 1914 and World War I broke out. He served as the head of the dermatology department at Edirne Military Hospital during the war between 1914 and 1918. Afterwards, he travelled abroad to further his medical knowledge. Following his initial visit to Budapest, he proceeded to Charite Hospital in Berlin. He had the chance to encounter highly skilled colleagues in his area and enhance his knowledge. He returned to Turkey after being in Europe between 1918 and 1919. Later, the Republic of Türkiye was established in 1923. On that date, he married Refika Hanım, the sister of one of his patients and the daughter of Suat Bey, a famous diplomat who was once an ambassador to Paris. Hulusi Behçet had a daughter named Güler.

In those days, Turkey experienced a continuous wave of innovation and reform. Emphasis was placed on scientific studies and efforts were made to keep up with modern societies. Dr. Behçet founded the Department of Dermatology and Venereal Diseases during this period, which laid the foundations of Dermatology in Turkey. He is the first person to receive the title of professor in the Republic of Türkiye [1]. Dr. Behçet was a highly productive scholar who authored over 100 articles presented at various national and international conferences. He was also renowned for his frequent journeys to establish academic partnerships [2].

In 1922, he became interested in syphilis and conducted some studies on the diagnosis and treatment of this disease. A year later, in 1923, he began his studies on Leishmaniasis disease. He was the first person to describe the nail sign appearing from the removal of the crust of an oriental sore.

Discovery of Behcet's disease

**Figure 1:**
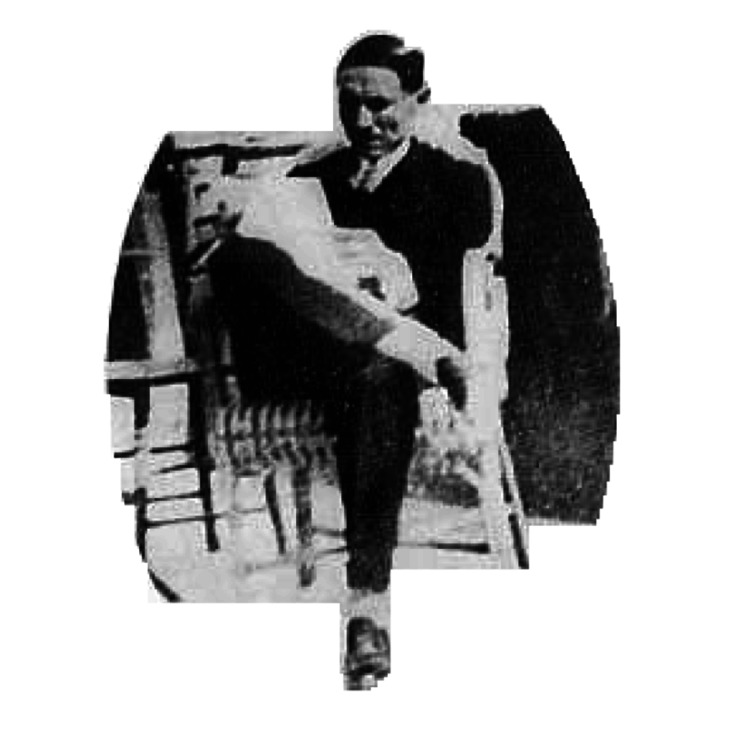
Among the records of a congress in Nice in 1934, reads even while resting. Credit: Permission for use was obtained from Istanbul University, Istanbul Medicine Faculty, Dermatology Department.

Hulusi Behçet observed Behçet's disease, which later became known by his name, between 1924 and 1925, due to a patient who had not been diagnosed for more than 40 years [1,3]. The patient had recurrent aphthous stomatitis, genital ulceration, erythema nodosum, and visual disturbances. The cause of these illnesses was previously thought to be syphilis and tuberculosis. However, Behçet thought that this could be due to a single aetiology. Five years after doing this study in 1930, he encountered another patient with similar symptoms. This patient had also oral and genital ulcerations and visual disturbance. In 1936, Behçet conducted a medical examination on a patient who presented symptoms of eye irritation, oral and scrotal ulcers, evening fever, and abdominal pain. By synthesizing the symptoms, Behçet hypothesized that they were diverse indications of a distinct illness, potentially infection caused by a virus. In 1937, he documented these cases in the Archives of Dermatology and Venereal Disease. Physicians from other countries like Belgium, Italy, Austria, the United States, Japan, Denmark, Switzerland, and Israel rapidly started reporting similar cases. Behçet's discoveries were officially recognized as pathognomonic of a novel disease at the International Congress of Dermatology in Geneva on September 13, 1947. Dr. Miescher, a professor of dermatology at the University of Zurich Medical School, suggested that the condition should be designated as "Morbus Behçet" (Behçet's disease) (Figure 1) [4].

Hulusi Behçet has authored more than 100 publications in the medical literature of Turkish, English, German, and French languages, and has also delivered several lectures. In addition, he authored a book titled "Diagnosis of Syphilis and Other Skin Disorders in Clinic and Practice". In 1936, he was appointed to the editorial boards of the journals *Dermatologische Wochenschrift* and *Medizinische Welt*. In 1939, the University of Istanbul granted him the prestigious title of "Ordinarius Professor," which is often bestowed upon academics to recognize their significant accomplishments throughout their career. In addition to Behçet's syndrome, Professor Behçet had expertise in diseases such as cutaneous leishmaniasis [5].

Behçet’s disease

**Figure 2:**
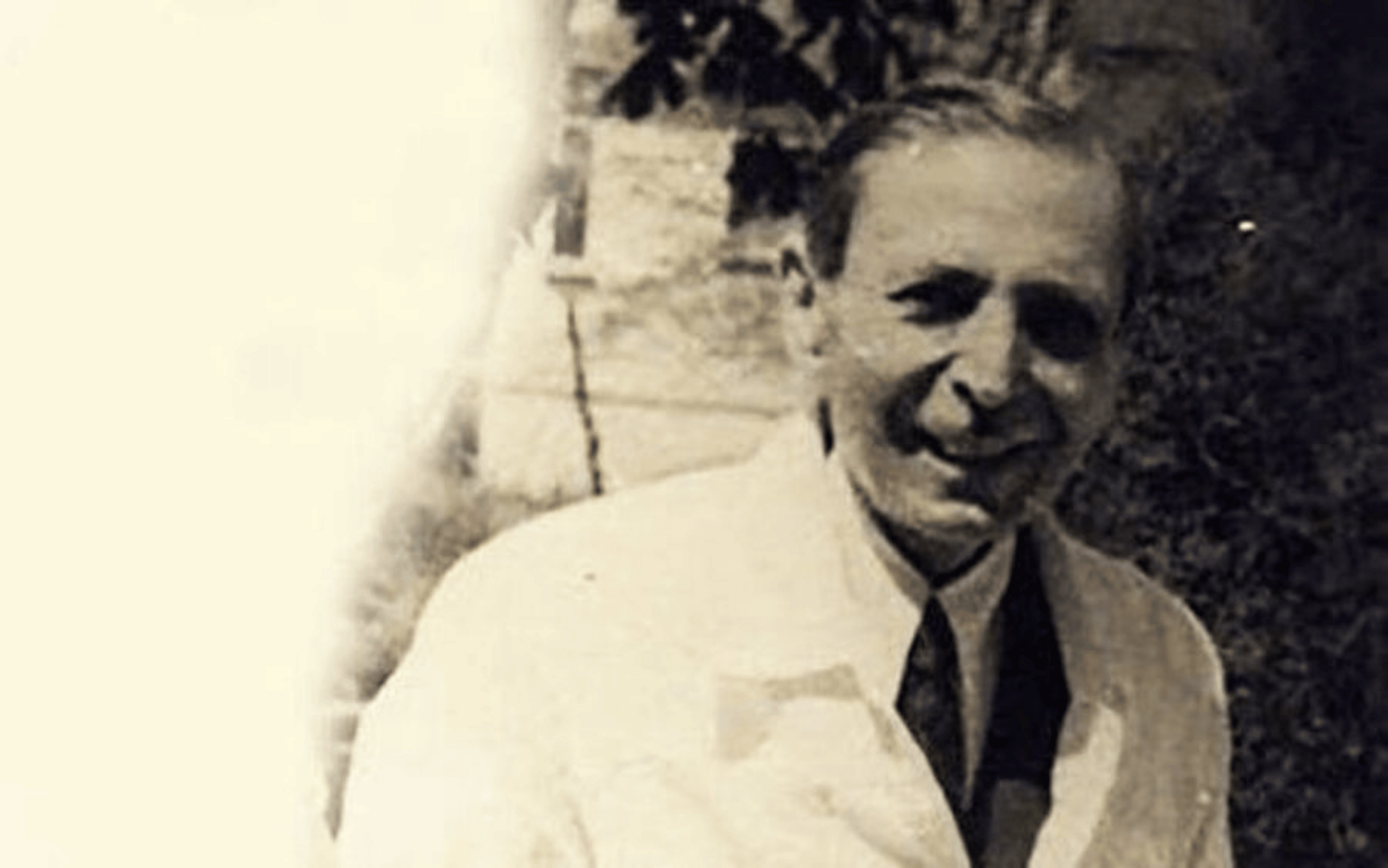
Hulusi Behcet at Istanbul University. Credit: Permission for use was obtained from Istanbul University, Istanbul Medicine Faculty, Dermatology Department.

Behçet’s disease is a unique disease characterized by recurrent aphthous stomatitis, uveitis, genital ulcers, and skin lesions. It typically impacts individuals between the ages of 20 and 40 years and is to a lesser extent observed in children [6]. Behçet's disease is more prevalent in countries located along the historical trade route known as the "Silk Road", which stretches from Japan to the Mediterranean region. Several studies have presented data indicating a robust association between human leukocyte antigen B51 (HLA-B51) and the disease across several ethnic groups [7]. The involvement of HLA-B51 in the disease has been a subject of discussion, as it may be linked to a nearby gene [8]. This is because the presence of HLA-B51 in patients with Behçet's disease is only around 60% [9]. Vasculo-Behçet's disease is the term used to describe the involvement of veins and arteries in Behçet's disease. In patients with Behçet's disease, venous thrombosis is the primary form of vascular involvement, occurring in 7-33% of cases. It causes 85-93% of vasculo-Behçet's disease [10]. The occurrence of deep vein thrombosis was found to have a strong correlation with being male and having a positive pathergy test [11]. In 1990, the International Study Group for Behcet’s Disease published an article for the diagnosis of Behçet's disease [12]. The three major signs of disease, which are oral aphthae, genital ulcer, and recurrent uveitis, were identified by Hulusi Behçet. Behçet's disease is treated using immunosuppressive drugs such as corticosteroids and colchicine. Although effective in reducing acute inflammation, corticosteroids by themselves generally prove ineffective in preventing relapses, so they are commonly used in combination with other drugs. Combination therapy is also employed to reduce the dosage of corticosteroids [13]. Also, azathioprine, cyclophosphamide, methotrexate, and interferon alpha are used in the treatment of disease [13]. Behçet's disease is Hulusi Behçet's most important contribution to medical science (Figure 2). Adamantiades, a Greek physician, published a clinical report of the condition in a French journal six years before Behçet's description [14]. The initially designated name for the disease was Adamantiades-Behçet's disease. However, the International Society for Behçet's Disease recommends using the word "Behçet's Disease".

Achievements and legacy

Dr. Hulusi Behçet's work did not stop with his identification of the disease. He continued to investigate and document the clinical aspects of the condition, and his research contributed to the development of diagnostic criteria and treatment approaches. His work has had a profound influence on the fields of dermatology, rheumatology, and internal medicine.

Behçet's contributions were formally recognized in the medical community, and his work remains a cornerstone of the study of autoimmune diseases. His identification of Behçet's disease has led to significant advancements in understanding and managing the condition, improving the quality of life for countless patients.

**Figure 3:**
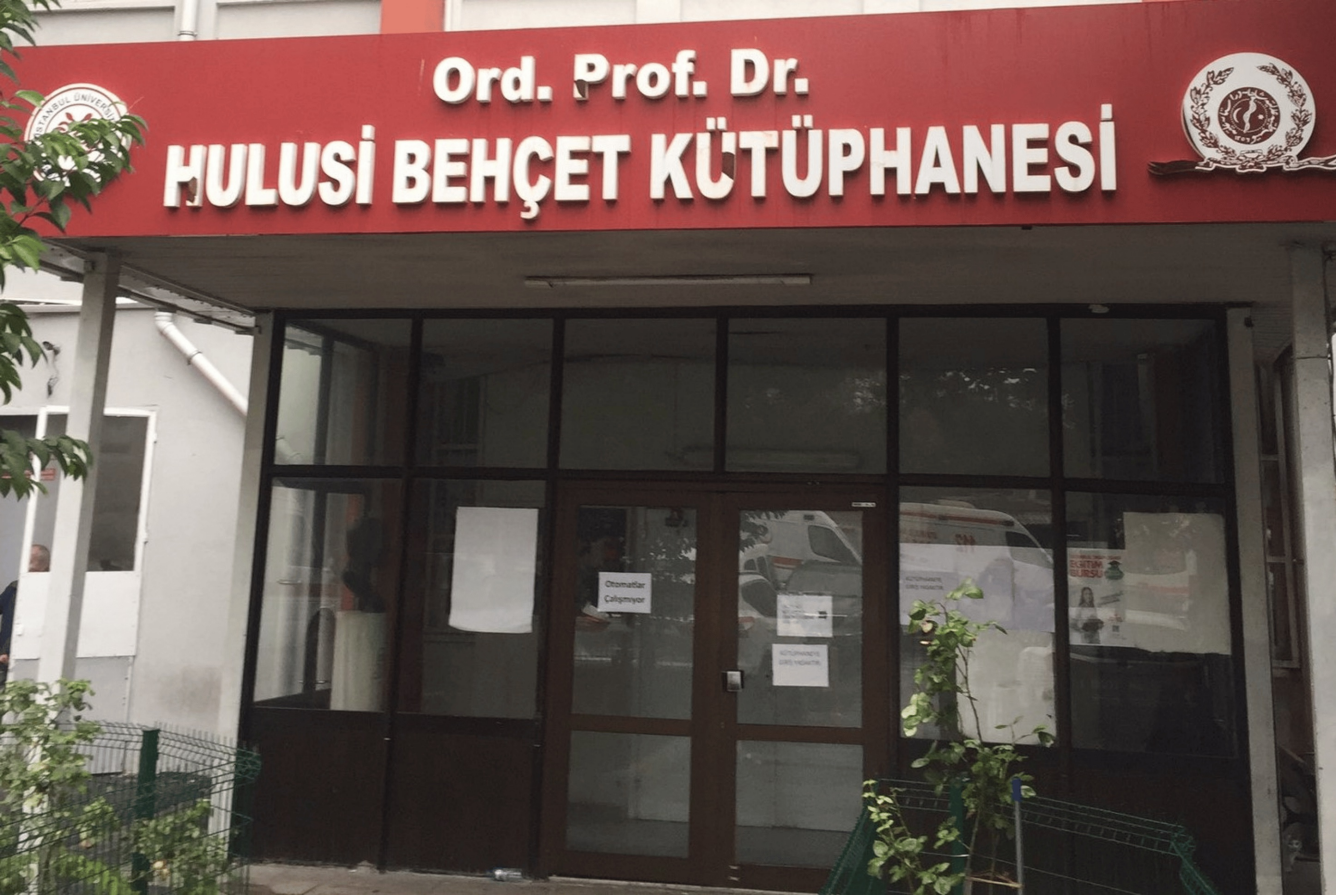
Ord. Prof. Dr. Hulusi Behcet Library in Istanbul University. Credit: Permission for use was obtained from Istanbul University, Istanbul Medicine Faculty, Deanery.

Dr. Behçet passed away on March 8, 1948, but his legacy endures. His name is commemorated in the eponymous Behçet's disease, a testament to his pioneering work and the impact he had on the field of medicine. Research into Behçet’s disease continues to build on his foundational work, exploring new treatments and a better understanding of the condition. Many years after his death, in 1975, he was awarded the TUBITAK Scientific Award. Many classes, libraries, and laboratories are named after him, for example, the big library at Istanbul University Medicine Faculty is named Behçet Kütüphanesi (Figure 3).

## Conclusions

Dr. Hulusi Behçet’s contributions to medicine, particularly in the identification and characterization of Behçet's disease, have had a lasting impact on medical science. His meticulous observations and pioneering research have paved the way for further advancements in the diagnosis and treatment of autoimmune disorders. Dr. Behçet’s legacy is a testament to the importance of dedication, curiosity, and innovation in advancing medical knowledge and improving patient care.
